# Reliability of measuring abductor hallucis muscle parameters using two different diagnostic ultrasound machines

**DOI:** 10.1186/1757-1146-2-33

**Published:** 2009-11-16

**Authors:** Wayne A Hing, Keith Rome, Alyse FM Cameron

**Affiliations:** 1 School of Rehabilitation & Occupation Studies, Health & Rehabilitation Research Centre, AUT University, Private Bag 92006, Auckland, 1142, New Zealand; 2 School of Rehabilitation & Occupation Studies, Health & Rehabilitation Research Centre, Discipline of Podiatry, AUT University, Private Bag 92006, Auckland, 1142, New Zealand

## Abstract

**Background:**

Diagnostic ultrasound provides a method of analysing soft tissue structures of the musculoskeletal system effectively and reliably. The aim of this study was to evaluate within and between session reliability of measuring muscle dorso-plantar thickness, medio-lateral length and cross-sectional area, of the abductor hallucis muscle using two different ultrasound machines, a higher end Philips HD11 Ultrasound machine and clinically orientated Chison 8300 Deluxe Digital Portable Ultrasound System.

**Methods:**

The abductor hallucis muscle of both the left and right feet of thirty asymptomatic participants was imaged and then measured using both ultrasound machines. Interclass correlation coefficients (ICC) with 95% confidence intervals (CI) were used to calculate both within and between session intra-tester reliability. Standard error of the measurement (SEM) calculations were undertaken to assess difference between the actual measured score across trials and the smallest real difference (SRD) was calculated from the SEM to indicate the degree of change that would exceed the expected trial to trial variability.

**Results:**

The ICCs, SEM and SRD for dorso-plantar thickness and medial-lateral length were shown to have excellent to high within and between-session reliability for both ultrasound machines. The between-session reliability indices for cross-sectional area were acceptable for both ultrasound machines.

**Conclusion:**

The results of the current study suggest that regardless of the type ultrasound machine, intra-tester reliability for the measurement the abductor hallucis muscle parameters is very high.

## Introduction

The widespread interest in the use of ultrasound (US) imaging in the musculoskeletal area over the last decade has lead to improvements in technology and the development of smaller less expensive machines with improved resolution [1]. US has also been reported to be a cost-effective and highly feasible method, among the imaging modalities, to measure muscle dorso-plantar thickness, medio-lateral width and cross-sectional area of muscles [2,3].

With the improved availability of US equipment there has been an increase in research evaluating intrinsic muscle parameters of the foot such as abductor hallucis, extensor digitorum brevis, the first interosseous dorsalis muscle, adductor hallucis and the first lumbrical muscle [4,5]. The abductor hallucis has been reported to play an important role in stabilising the medial longitudinal arch and is necessary for efficient toe-off in late stance of walking. The abductor hallucis is one of the muscles that has also been reported to be adversely affected in hallux valgus and in diabetic neuropathy. Understanding muscle parameters of the abductor hallucis may change the way practitioners manage pes planus, posterior tibial tendon dysfunction, hallux valgus, and Charcot neuroarthropathy [5-9]. However, the need to undertake reliable measurements is a necessary requirement before any interventions or management plans are undertaken.

With the advent of smaller transducers and more portable US machines, the ability to evaluate smaller joints and muscles has made US more user-friendly in the routine clinical care setting [1]. With the introduction of new technology, there is a need to identify the reliability of measuring muscle parameters such as thickness and cross-sectional area. Other important issues in the musculoskeletal ultrasound arena are the assessment of inter-scanner variability [1] and inter-machine variability [10]. A previous study reported on the excellent intra-tester reliability of using one US machine to assess abductor hallucis muscle parameters [4]. However, clinicians should be aware of measurement errors involved when using different US machines. This is of particular importance when there is inter-changeability of US machines in large clinical settings. Therefore, the aim of the study was to evaluate within- and between-session reliability of measuring muscle dorso-plantar thickness, medio-lateral length and cross-sectional area of the abductor hallucis muscle using two different US machines commonly used in different scopes of clinical practice.

## Methods

### Participants

Thirty participants (twenty female, ten male), recruited from the general University population, completed the study with a mean age of 28.24 ± 10.2 years, mean weight of 68.8 ± 12.35 Kg, and a mean height of 1.71 ± 0.97 m. Informed consent to participate in this study was given by all participants. Participants met the inclusion criteria if they were healthy individuals between the ages 18-60 and did not have a history of inflammatory arthritis, previous foot or ankle surgery, diabetes, lower limb amputation, or severe hallux valgus as defined by the Manchester Scale [11]. The University Ethics Committee approved the procedures used in this study.

### Equipment

A 'higher end' Philips HD11 ultrasound machine, with linear transducer (12-5 MHz), and a Chison 8300 Deluxe Digital 'portable' ultrasound system, with linear transducer (7.5 MHz) were used to scan images of the abductor hallucis muscle. An Aquaflex^® ^Ultrasound Gel Pad (Fairfield, USA) was applied directly onto the participant's skin, over the abductor hallucis muscle, ensuring optimal transducer contact and signal penetration. Philips Q-lab Software (Release 5.0) was employed for data quantification from the images taken from the Philips HD11, and the Chison 8300 inbuilt software was used for the images captured on that machine.

### Experimental Procedure

The abductor hallucis muscle of both the left and right foot, for each of the thirty participants were imaged, for digital investigation, and three separate images of the medio-lateral thickness, dorso-plantar thickness and cross-sectional area of each foot were recorded. The transverse plane of the abductor hallucis muscle was utilised in the current study as preliminary pilot work had identified the transverse plane to be the best plane to assess the muscle.

This experimental procedure was undertaken with the same participant on both the Philips HD11 and Chison 8300 for between machine reliability. This entire process was then repeated at least three days post (mean = 8.7 days) to obtain between day test results. Ultrasound imaging and measurements were performed by one sonographer of 2 years experience.

**Figure 1:**
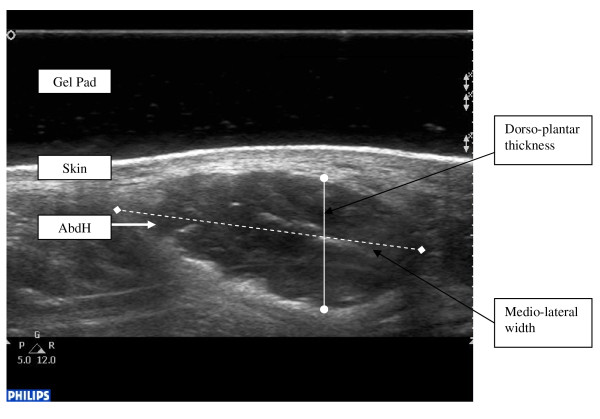
**Ultrasound image of abductor hallucis muscle with dorso-plantar thickness and medio-lateral width points marked from Phillips HD11(longitudinal view)**.

**Figure 2:**
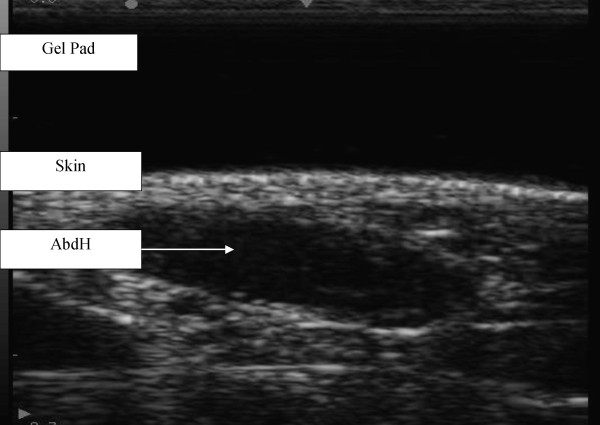
**Ultrasound image of abductor hallucis muscle from Chison 8300 (longitudinal view)**.

Each participant was positioned in supine lying. The heel and plantar aspect, excluding the first metatarsal, of the involved foot rested against a stable platform designed to fix the ankle in a zero degree neutral position. The posterior aspect of the knee was supported in approximately 15-degrees flexion. The uninvolved leg was also supported. The sonographer manually palpated relevant bony anatomical landmarks and marked them for orientation. These included a reference line for scanning directly inferior from the most anterior aspect of the medial malleolus. Scanning occurred with the transducer applied onto the gel pad that lay on the skin overlying the abductor hallucis muscle belly at a perpendicular angle to the aforementioned scanning line and long axis of the foot on the proximal aspect of the reference line to encompass the muscle fibres of abductor hallucis. The abductor hallucis muscle was imaged with the transducer applied at a perpendicular angle to the long axis of the foot on the proximal aspect of the reference line. Minimal pressure was applied with the transducer to reduce any possible alterations to the muscle fibres and architecture. Three separate images of the abductor hallucis muscle were captured using both machines for both left and right feet (Figure 1 &2) and stored on the hard drive for later analysis. Measurement and analysis were undertaken independent of one another to ensure blinding of the results. Blinding was undertaken by identifying and removing all identifiable details such as image number, US machine and patient number and the sonographer randomly evaluated each of the scanned images.

The Philips HD11 images were analysed using onscreen digital callipers, where as the Chison 8300 measurements were calculated using manual measurement and scale. The dorso-plantar thickness of the abductor hallucis muscle was measured at the widest thickness measurement, perpendicular from the most inferior aspect of the muscle belly to the most superior point of the muscle. The medio-lateral width of the abductor hallucis was measured from the most superficial border to the deepest border also using digital callipers. The muscle cross-sectional area measurement of the abductor hallucis muscle was gained through the digital manual tracing, a built in feature of each of the US machines, of the muscle borders for both diagnostic US machines.

### Data Analysis

The baseline descriptive information obtained from each participant was stored for statistical analysis. An analysis of statistical comparisons of within-session reliability, between-session reliability - single measures, between-session reliability - average measures reliability of the two ultrasound imaging machines was carried out using SPSS (version 15, SPSS Inc., Chicago, IL). Repeated measures (test-retest) reliability analyses utilised Interclass Correlation Coefficients (ICC, 3.1) and 95% confidence intervals (CI). It has been previously reported that > 0.90 = excellent, > 0.80-0.89 = high, and >0.70-0.80 = acceptable [12]. As with other reliability coefficients, there is no standard acceptable level of reliability using the ICC [13]. It is stated that any measure should have an ICC of at least 0.6 to be useful [14]. Bland-Altman plots have been used to provide graphical representation of key reliability findings [15,16]. The Bland-Altman method calculates the range within which the difference between the two occasions will lie with a probability of 95% [15,16].

Standard error of the measurement (SEM) calculations were undertaken to assess difference between the actual measured score across trials and an estimated "true" score [17,18]. The smallest real difference (SRD) was calculated from the SEM that indicates the degree of change that would exceed the expected trial to trial variability [18,19].

## Results

Descriptive information of the abductor hallucis muscle medio-lateral width, dorso-plantar thickness and cross-sectional area for both US machines are presented in Table 1.

**Table 1:** Descriptive statistics of abductor hallucis muscle parameters

		Phillips HD11	Chison 8300
	Day	Mean ± SD	Mean ± SD
Dorso-Plantar thickness (mm)	1	11.56 ± 1.07	11.54 ± 1.13
	2	11.54 ± 1.02	11.60 ± 1.07
Medio-lateral width (mm)	1	28.98 ± 2.77	28.81 ± 2.75
	2	29.03 ± 2.60	28.97 ± 2.62
Cross-sectional area (mm^2^)	1	269.33 ± 35.99	262.35 ± 38.60
	2	275.99 ± 36.04	270.26 ± 34.31

### Within Session Reliability

The results for within-session reliability analysis, for both the Philips and Chison ultrasound machines, demonstrated excellent reliability for the three abductor hallucis muscle parameters measured (Table 2). The low SEM and within-session SRD values for dorso-plantar thickness, medio-lateral width and cross-sectional area measurements also indicate low measurement error for both the Philips HD11 and Chison 8300 (Table 2).

**Table 2:** Within session reliability

	ICC [3.1]	95% CI	SEM	SRD
**Phillips HD11**				

Dorso-plantar thickness (mm)	0.97	(0.95 to 0.99)	0.12	0.34

Medio-lateral width (mm)	0.96	(0.95 to 0.98)	0.87	2.47

Cross-sectional area (mm^2^)	0.98	(0.96 to 0.98)	9.36	26.59

				

**Chison 8300**				

Dorso-planatr thickness (mm)	0.99	(0.99 to 0.99)	0.09	0.26

Medio-lateral length (mm)	0.95	(0.92 to 0.97)	1.06	3.01

Cross-sectional area (mm^2^)	0.99	(0.98 to 0.99)	6.73	19.12

### Between Session Reliability - Single measures

Single measures analysis illustrated excellent reliability for dorso-plantar thickness measurements of abductor hallucis for both the Philips HD11 and Chison 8300 (Table 3). Medio-lateral width measurements were deemed of high reliability for the Phillips HD11 and acceptable for the Chison 8300 (Table 3). Reliability for cross-sectional area measurements were below the acceptable level for both machines (Table 3). Low SEM values for both dorso-plantar thickness and medio-lateral width again indicate a low level of measurement error for both US imaging machines (Table 3). The SEM between-session single measure for cross-sectional area were considerably high when compared to the within session values (Table 2 &3).

**Table 3:** Between session reliability: Single measures

	ICC [3.1]	95% CI	SEM	SRD
**Phillips HD11**				

Dorso-plantar thickness (mm)	0.95	(0.91 to 0.97)	0.46	1.31

Medio-lateral width (mm)	0.88	(0.81 to 0.93)	0.28	0.79

Cross-sectional area (mm^2^)	0.65	(0.48 to 0.78)	40.10	113.93

**Chison 8300**				

Dorso-plantar thickness (mm)	0.92	(0.87 to 0.95)	0.13	0.37

Medio-lateral width (mm)	0.78	(0.66 to 0.87)	0.08	0.22

Cross-sectional area (mm^2^)	0.64	(0.47 to 0.77)	35.30	100.29

### Between Session Reliability -Average measures

By taking the average of three measures an excellent reliability of measuring the dorso-plantar thickness of abductor hallucis, for both machines was obtained (Table 4). An excellent (Philips HD11) and high (Chison 8300) reliability was found for the measuring medio-lateral width (Table 4). Cross-sectional area between-session reliability measurements were acceptable for both US machines (Tables 4). The SEM and SRD values for dorso-plantar thickness, medio-lateral width and cross-sectional area again consistently showed a low level of measurement error (Table 4).

**Table 4:** Between session reliability: average measures

	ICC [3.1]	95% CI	SEM	SRD
**Phillips HD11**				

Dorso-plantar thickness (mm)	0.97	(0.95 to 0.98)	0.25	0.71

Medio-lateral width (mm)	0.94	(0.90 to 0.96)	0.90	2.56

Cross-sectional area (mm^2^)	0.79	(0.65 to 0.88)	20.15	57.24

**Chison 8300**				

Dorso-plantar thickness (mm)	0.96	(0.93 to 0.97)	0.32	0.91

Medio-lateral width (mm)	0.88	(0.80 to 0.93)	1.20	3.41

Cross-sectional area (mm^2^)	0.78	(0.64 to 0.87)	21.78	61.88

**Figure 3:**
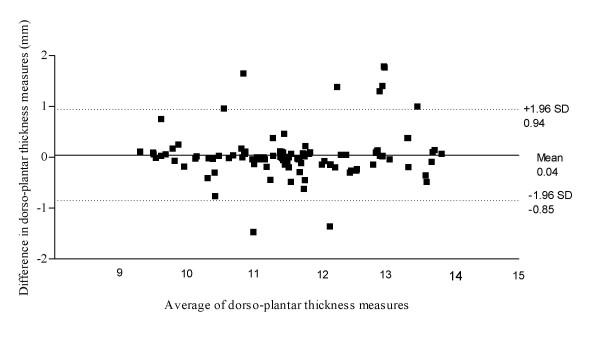
**Bland & Altman plot for Phillips HD11 and Chison 8300 results for abductor hallucis Dorso-plantar thickness**.

**Figure 4:**
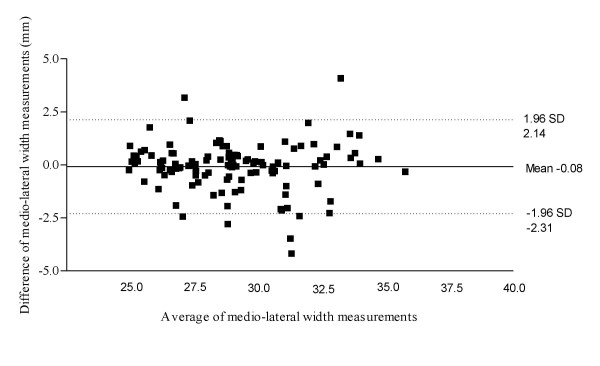
**Bland & Altman plot for Phillips HD11 and Chison 8300 results for abductor hallucis medio-lateral width**.

**Figure 5:**
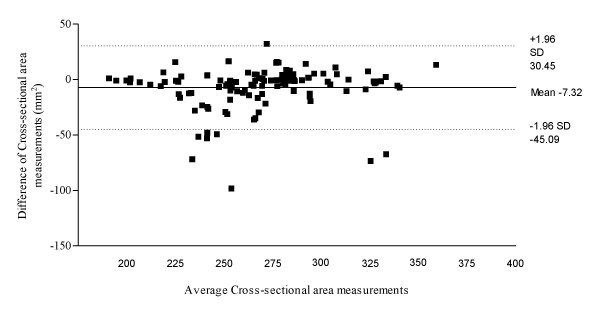
**Bland & Altman plot for Phillips HD11 and Chison 8300 results for abductor hallucis cross-sectional area**.

Figure 3 illustrates the Bland & Altman plot for Philips HD11 and Chison 8300 within session results for abductor hallucis dorso-plantar thickness, with a 95% limits of agreement, bias of 0.04 with a SD of bias of 0.46 (lower limit -0.85, upper limit 0.94). Figure 4 illustrates the Bland & Altman plot for Philips HD11 and Chison 8300 within session results for abductor hallucis medio-lateral width, with a 95% limits of agreement, bias of -0.08 with a SD of bias of 1.14 (lower limit -2.31, upper limit 2.14). Figure 5 illustrates the Bland & Altman plot for Philips HD11 and Chison 8300 within session results for abductor hallucis cross-sectional area, with a 95% limits of agreement, bias of -7.32 with a SD of bias of 19.27 (lower limit -45.09, upper limit 30.45).

## Discussion

The results of the current study demonstrated that diagnostic US is an effective and reliable tool for measuring abductor hallucis muscle parameters. To the authors knowledge there has been no previous research investigating intrinsic muscle structure using different US machines, therefore the current SRD reflect the potential to detect changes that exceed measurement error for research application.

Overall, Philips HD11 and Chison 8300 show excellent to high reliability in measuring dorso-plantar thickness and medio-lateral width using ICCs, SEM and SRD, indicating the potential to utilise these two parameters in order to follow the progression or change of the muscle's architecture. Both US machines demonstrated acceptable between-session reliability when measuring the cross-sectional area; however, the SEM and SRD values attained may be secondary to the human error influence of manual tracing of the abductor hallucis muscle borders. Furthermore, the SRD demonstrated higher measurement error when only one measure compared to three measures to obtain a mean measurement. Clinicians should consider obtaining three measures rather than a single measure. Digital/computer generated mapping of the muscles could be a possibility in future research for evaluating cross-sectional area. Reeves et al [20] suggest that measurement error can be reduced by comparing US cross-sectional results to MRI images in order to assure the accuracy of the cross-sectional area. However, this is a costly method to adopt in the clinical setting. Although MRI can be more precise than US, it also has limitations and is dependent on the resolution of the machine. Future studies could be undertaken to compare gold standards such as MRI or CT scans against US to evaluate intrinsic muscle parameters.

The research implications of this study include the potential of utilising the affordable, portable US machines in the clinical environment more regularly. Ultrasonography has the potential to be employed to further investigate and undertake measurement analyses of individuals with foot and ankle pathologies. The cost of US equipment has been stated to directly relate to the attained images resolution and quality; this therefore indicates that a high-resolution ultrasonography machine produces higher quality images, which are more easily interpreted [21]. However, the reliability of the results of this research indicate that images gained and analysed from both the more costly Philips HD11, and less expensive Chison 8300 are very similar in comparison, therefore indicating that it may not be of immediate importance to which machine is used in order to establish basic muscle anatomical parameters. Although, caution needs to be applied if the two US machines are used interchangeably in practice in order to gain accurate results.

A low SEM value relative to the resting value would imply an ability to detect a real change, without being influenced by measurement error. The smallest real difference (SRD) can be calculated to indicate the degree of change that would exceed the expected trial to trial variability [17,19].

In addition to the findings of excellent within session and average between session reliability for a single assessor, the reported SEM values were low when compared to the resting dorso-plantar thickness and medio-lateral width values of the muscle. This may have clinical importance, with a likely application being the measurement of thickness or width due to muscle contraction or pathology. A low SEM value relative to the resting value would suggest that the ability to detect a real change (exceeding measurement error) would be likely. With respect to between session average reliability, based on the SEM of 0.25 mm for the abductor hallucis muscle, dorso-plantar thickness SRD measurement can be calculated by the following formula: *SEM *× √2 × 2.009 (where 2.009 represents the *t *value of distribution for a 95% CI (*df *= 59). If this value is divided by the average dorso-plantar thickness of the muscle (11.56 mm) a change in thickness of greater than 3.0% would be required to be 95% confident that a real change has occurred. Further, with regard to medio-lateral width and cross-sectional area, a change greater than 8.8% and 21.25% respectively would be required to be 95% confident that a real change occurred. The higher percentage seen in cross-sectional area estimation could be due to the added human error possibility in the manual measuring the cross-sectional area.

## Conclusion

In summary, the results of the current study only tested the reliability of two types of US machines but found intra-tester reliability for the measurement the abductor hallucis muscle parameters to be high. The results from the current work using US imaging to measure the muscle parameters of dorso-plantar thickness, medio-lateral width and cross-sectional area of the abductor hallucis muscle suggest excellent to high within-session reliability for both US machines, although between-session reliability for cross-sectional measurements was acceptable.

## Competing interests

The authors declare that they have no competing interests.

## Authors' contributions

KR and WH conceived and designed the study. AC collected and inputted the data. KR, WH and AC conducted the statistical analysis. KR and WH compiled the data and drafted the manuscript and AC contributed to the drafting of the manuscript. All authors read and approved the final manuscript.

## References

[B1] Conaghan PG Musculoskeletal ultrasonography: Improving our senses Arthritis Care Res 2005 53 639 642 10.1002/art.21448 16208673

[B2] Norasteh A Ebrahimi E Salavati M Rafiei J Abbasnejad E Reliability of B-mode ultrasonography for abdominal muscles in asymptomatic and patients with acute low back pain J Bodyw Mov Ther 2007 11 17 20 10.1016/j.jbmt.2005.11.002

[B3] Koppenhaver SL Hebert JJ Fritz JM Parent EC Teyhen DS Magel JS Reliability of rehabilitative ultrasound imaging of the transversus abdominis and lumbar multifidus muscles Arch Phys Med Rehabil 2009 90 87 94 10.1016/j.apmr.2008.06.022 19154834

[B4] Cameron A Rome K Hing WA Ultrasound evaluation of the abductor hallucis muscle: Reliability study J Foot Ankle Res 2008 1 12 10.1186/1757-1146-1-12 18822116 PMC2565658

[B5] Severinsen K Andersen H Evaluation of atrophy of foot muscles in diabetic neuropathy - a comparative study of nerve conduction studies and ultrasonography J Clin Neurophysiol 2007 118 2172 175 10.1016/j.clinph.2007.06.019 17709290

[B6] Arinci Incel N Genc H Erdem HR Yorgancioglu ZR Muscle imbalance in hallux valgus: an electromyographic study Am J Phys Med Rehabil 2003 82 345 9 10.1097/00002060-200305000-00003 12704272

[B7] Brenner E Insertion of the abductor hallucis muscle in feet with and without hallux valgus Anat Rec 1999 254 429 34 10.1002/(SICI)1097-0185(19990301)254:3<429::AID-AR14>3.0.CO;2-5 10096675

[B8] Greenman RL Khaodhiar L Lima C Dinh T Giurini JM Veves A Foot small muscle atrophy is present before the detection of clinical neuropathy Diabetes Care 2005 28 1425 30 10.2337/diacare.28.6.1425 15920063 PMC1224714

[B9] Wong YS Influence of the abductor hallucis muscle on the medial arch of the foot: a kinematic and anatomical cadaver study Foot Ankle Int 2007 28 617 20 10.3113/FAI.2007.0617 17559771

[B10] Wakefield RJ D'Agostino M-A Iagnocco A Filippucci E Backhaus M Scheel AK Joshua F Naredo E Schmidt WA Grassi W Moller I Pineda C Klauser A Szkudlarek M Terslev L Balint P Bruyn GAW Swen WAA Jousse-Joulin S Kane D Koski JM O'Connor P Milutinovic S Conaghan P Group OU The OMERACT Ultrasound Group: status of current activities and research directions J Rheumatol 2007 34 848 51 17407236

[B11] Garrow AP Papgeorgiou A Silman AJ Thomas E Jayson MIV Macfarlane GJ The grading of the hallux valgus, the Manchester Scale J Am Podiatr Med Assoc 2001 91 74 78 11266481 10.7547/87507315-91-2-74

[B12] Vincent WJ Statistics in kinesiology 1999 Leeds: Human Kinetics

[B13] Bruton A Conway JH Holgate ST Reliability: what is it, and how is it measured? Physiotherapy 2000 86 94 95 10.1016/S0031-9406(05)61211-4

[B14] Rankin G Stokes M Reliability of assessment tools in rehabilitation: an illustration of appropriate statistical analyses Clin Rehabil 1998 12 197 199 10.1191/026921598672178340 9688034

[B15] Mantha S Roizen M Fleisher L Thisted R Foss J Comparing methods of clinical measurement: reporting standards for Bland and Altman analysis Anesth Analg 2000 90 593 602 10.1097/00000539-200003000-00018 10702443

[B16] Bland J Altman D Applying the right statistics: analyses of measurement studies Ultrasound Obstet Gynecol 2003 22 85 93 10.1002/uog.122 12858311

[B17] Wallwork TL Hides JA Stanton WR Intrarater and interrater reliability of assessment of lumbar multifidus muscle thickness using rehabilitative ultrasound imaging J Orthop Sports Phys Ther 2007 37 608 612 17970407 10.2519/jospt.2007.2418

[B18] McPoil T Vicenzino B Cornwall M Collins N Warren M Reliability and normative values for the foot mobility magnitude: a composite measure of vertical and medial-lateral mobility of the midfoot J Foot Ankle Res 2009 2 6 10.1186/1757-1146-2-6 19267907 PMC2656480

[B19] Ota S Ward SR Chen YJ Tsai YJ Powers CM Concurrent criterion-related validity and reliability of a clinical device used to assess lateral patellar displacement J Orthop Sports Phys Ther 2006 36 645 652 17017269 10.2519/jospt.2006.2263

[B20] Reeves ND Maganaris CN Narici MV Ultrasonographic assessment of human skeletal muscle size Eur J Appl Physiol 2004 91-1 116 118 10.1007/s00421-003-0961-9 14639480

[B21] Kane D Grassi W Sturrock R Balint PV A brief history of musculoskeletal ultrasound: 'From bats and ships to babies and hips' Rheumatology 2004 43 931 933 10.1093/rheumatology/keh004 15213339

